# Prognostic Factors of COVID-19 Infection in Elderly Patients: A Multicenter Study

**DOI:** 10.3390/jcm9123932

**Published:** 2020-12-04

**Authors:** Jihye Hwang, Ho-Sung Ryu, Hyun Ah Kim, Miri Hyun, Ji Yeon Lee, Hyon-Ah Yi

**Affiliations:** 1 Department of Neurology, Keimyung University Daegu Dongsan Hospital, Daegu 41931, Korea; jhhwang0110@gmail.com; 2 Department of Neurology, Kyungpook National University Hospital, Daegu 41944, Korea; ryuhosung138@gmail.com; 3 Department of Medicine, Division of Infectious Diseases, Keimyung University Dongsan Hospital, Keimyung University School of Medicine, Daegu 42601, Korea; hyunah1118@dsmc.or.kr (H.A.K.); eternity7919@dsmc.or.kr (M.H.); 4 Department of Neurology, Keimyung University Dongsan Hospital, Keimyung University School of Medicine, Daegu 42601, Korea

**Keywords:** COVID-19, SARS-CoV-2, novel coronavirus, elderly, prognosis

## Abstract

The outbreak of the COVID-19 pandemic is a substantial threat to the health of all populations worldwide, and old age is a robust risk factor for poor prognosis of COVID-19 infection. To reduce the fatality rate of COVID-19 infection, further understanding of elderly patients with COVID-19 is necessary. We aimed to investigate the prognostic factors in elderly patients with COVID-19. This was a multicenter and retrospective study. Overall, 340 elderly patients with COVID-19 were enrolled in 3 hospitals in Daegu, South Korea. Death and severe pneumonia requiring oxygen treatment were defined as poor clinical outcomes. Of the patients studied, 15% died and 35.2% were classified as having severe pneumonia. In binary logistic regression analysis, activities of daily living (ADL) impairment, fever during hospitalization, initial infiltration on chest radiograph, and initial increased C-reactive protein (CRP) were significantly associated with severe pneumonia (OR = 5.33, *p* < 0.001; OR = 3.2, *p* = 0.002; OR = 2.32, *p* = 0.044; and OR = 1.33, *p* < 0.001, respectively). ADL impairment, comorbidity, fever during hospitalization, and initial increased CRP were significantly associated with death (OR = 7.13, *p* < 0.001; OR = 3.28, *p* = 0.005; OR = 3.15, *p* = 0.032, and OR = 1.18, *p* < 0.001, respectively). ADL impairment, fever, and initial CRP were poor prognostic factors in elderly patients with COVID-19. Understanding these poor prognostic factors is necessary to control the disease in elderly patients.

## 1. Introduction

Coronavirus Disease 2019 (COVID-19) is caused by a novel RNA virus named severe acute respiratory syndrome coronavirus 2 (SARS-CoV-2, previously known as 2019-nCoV), which emerged in December 2019 in Wuhan, Hubei Province, China [1,2,3]. An estimated 82,000 confirmed cases of COVID-19 infection, including COVID-19 pneumonia, and more than 4600 deaths were reported in China in the early stages, and the number of cases has increased rapidly. As the disease spread rapidly around the world, the World Health Organization (WHO) declared a pandemic. As of 27 May 2020, 5,488,825 cases of COVID-19 had been reported globally according to the World Health Organization [4].

As of 5 June 2020, 11,668 patients had been diagnosed with COVID-19. To date, 23,889 patients have been confirmed with COVID-19 in South Korea [5]. During the first outbreak from February to May, 6884 patients had been diagnosed with COVID-19 in Daegu City, which has the largest number of total cases with COVID-19 in Korea, accounting for 30% the nation’s confirmed cases [6]. A total of 415 patients with COVID-19 have died in South Korea and the case-fatality ratio (CFR) is 1.74%. In Italy, which has had 22,512 confirmed cases and 1625 deaths, the CFR is 7.2% [7,8]. Meanwhile, in France, which has had 526,435 confirmed cases and 31,691 deaths, the CFR is 6.0%, and in New York City, which has had 244,757 confirmed cases and 23,823 deaths, the CFR is 9.7% [9,10]. The CFR in South Korea is substantially lower than those observed in other countries, which may be related to patient demographics, the capacity of the medical resource in infectious disease surge, and healthcare system quality.

Old age is a well-known risk factor for poor prognosis of COVID-19 infection [11,12]. The fatality rate gradually increases with age. Elderly patients between 60–69 years old have a CFR of 1.16% (44 deaths/3795 confirmed cases), patients between 70–79 years old have a CFR of 7.21% (138 deaths/1913 confirmed cases), and patients above 80 years old have CFR of 21.33% (208 deaths/975 confirmed cases) [5]. In Italy, elderly patients between 60–69 years old have a CFR of 3.5%, and patients above 80 years old have CFR of 20.2% (850 death/4209 confirmed cases) [8,13]. Elderly patients with COVID-19 infection require more intensive treatment and have higher severity and mortality rates than young patients with COVID-19. Old age is a robust risk factor for severe disease and death, as shown in previous studies [3,11,14].

In cases with underlying diseases, the elderly have been reported to be more susceptible to COVID-19 [15,16]. Some chronic diseases raise the risk of poor prognosis of COVID-19. In particular, diabetes is known as a major risk factor of COVID-19 [14,17]. However, it is not well known whether diabetes specifically increases the risk of COVID19 in elderly patients [18,19].

As COVID-19 spreads to communities around the world, outbreaks in nursing facilities have increased [20,21]. Outbreaks in nursing facilities are dangerous in that they can spread rapidly to local communities and to a large number of patients at once, resulting in quarantine and burdens on medical systems [22,23]. Additionally, patients in nursing facilities are mostly elderly individuals with many underlying diseases, likely increasing the severity of the disease.

A prognostic study of elderly patients with COVID-19 infection is needed to control COVID-19 infection, as the highest mortality rates are reported in elderly patients. It is important to understand the characteristics of elderly patients with COVID-19 infection to reduce the mortality of patients with COVID-19 and overcome the disease. The aim of this study was to investigate the prognostic factors of elderly patients with COVID-19 infection.

## 2. Materials and Methods

### 2.1. Study Design and Participants

This study was a retrospective, multicenter cohort study. We enrolled patients with COVID-19 who were admitted between 17 February 2020 and 31 May 2020 in Keimyung University Daegu Dongsan Hospital, Keimyung University Dongsan Hospital, and Kyungpook National University Hospital in Daegu City, South Korea. All patients were diagnosed with COVID-19 using real-time reverse-transcriptase-polymerase-chain-reaction (RT-PCR). We included only patients who were discharged or deceased by 5 June 2020 in three hospitals. Of the 792 patients who were discharged or deceased, only 340 elderly patients (≥65 years old) were included in this study. We retrospectively reviewed the electronic medical records of 340 elderly patients with COVID-19. Clinical outcomes were followed up until 5 June 2020.

### 2.2. Standard Protocol Approvals, Registrations, and Patient Consent

This study was approved by the local public institutional review board of Daegu (DGIRB 2020-08-001). This study was performed in accordance with the ethical standards of the 1964 Declaration of Helsinki and its later amendments or comparable ethical standards. The requirement for informed consent was waived because we only used deidentified data collected for clinical purposes during hospitalization.

### 2.3. Data Collection and Definition

Epidemiological, clinical, laboratory, and radiological characteristics; treatment; and outcome data were obtained with standardized data collection forms based on electronic medical records.

All patients with COVID-19 were diagnosed as positive using RT-PCR for SARS-CoV2 in nasopharyngeal and oropharyngeal swabs, regardless of symptoms, as a definition of the Korea Disease Control and Prevention Agency (KDCA). All confirmed patients with COVID-19 were admitted at hospitals or therapeutic living centers according to the severity of symptoms, underlying diseases, and medical requirements [24].

The patients were discharged if all of the following conditions were satisfied: (1) Asymptomatic, (2) did not use medication or recovering after taking medication for a sufficient period, (3) negative for RT-PCR twice in succession based on the guideline of the KDCA. Also, all of the deceased patients who had been diagnosed with COVID-19 were included in this study.

We classified patients into mild pneumonia and severe pneumonia, according to the interim guideline of WHO [25]. Following the WHO guideline, severe pneumonia is defined as having a fever or suspected respiratory infection, plus one of the following: Respiratory rate > 30 breaths/min, severe respiratory distress, or SpO_2_ ≤ 93% on room air. We also provided supplemental oxygen therapy to patients with severe pneumonia via nasal cannula, facial mask, or noninvasive or invasive mechanical ventilation in the case of respiratory distress, hypoxemia of low SpO_2_, or high respiratory rate. Based on the guideline, the timing and type of oxygen therapy were determined by the clinical decision of the physicians specialized in infectious disease or pulmonology.

We collected data on age, sex, the date of diagnosis, the date of symptom onset, days from diagnosis to admission, the date of admission, duration of hospitalization, discharge or deceased date, premorbid baseline activities of daily living (ADL) on admission, living in a nursing facility before admission, comorbidities (diabetes mellitus, hypertension, congestive heart failure, coronary arterial disease, lung disease such as asthma and chronic obstructive pulmonary disease, stroke, dementia, history of malignancy or current malignancy, chronic kidney disease, and liver disease), initial symptoms (such as cough, sputum, blood-tinged sputum, sore throat, rhinorrhea, chest pain, myalgia, fatigue, dyspnea, anosmia, headache, mental change, abdominal pain, diarrhea, nausea/vomiting), initial vital signs, the highest fever during hospitalization, antiviral treatment, antimalarial treatment, antibacterial treatment, steroid treatment, oxygen therapy, intensive care unit treatment, mechanical ventilation requirements, extracorporeal membrane oxygenation, and dialysis.

Total hospital days were defined as days from admission to discharge or date of death. Fever was defined as a temperature of 37.5 °C or higher during hospitalization. During hospitalization, body temperature was measured daily. Premorbid baseline ADL impairment was classified according to whether the patient could independently perform daily activities before being diagnosed. For the comorbidity score, diabetes mellitus, hypertension, heart disease, lung disease, chronic neurological disease history of malignancy or current malignancy, and chronic kidney disease were each given 1 point and classified as 2 points or more, 1 point, or 0 points.

Routine blood examination results were collected. Initial blood tests during hospitalization included complete blood count, serum biochemical tests (including renal and liver function, lactate dehydrogenase, and electrolytes), and coagulation profile. The date and results of chest radiographs or CT scans were also collected.

We defined the deceased group and the severe pneumonia group that received oxygen therapy during treatment as having a poor outcome. We compared the deceased group and the survival group and compared the patients with severe pneumonia requiring oxygen therapy and those who did not.

### 2.4. Statistical Analysis

We compared the deceased group and the survival group, as well as the severe pneumonia group requiring oxygen therapy and the mild pneumonia group. Demographic factors and patient characteristics were compared using Student’s t-test for continuous variables and χ^2^ or Fisher’s exact test for categorical variables. Analysis of covariance (ANCOVA) was performed to adjust for age between both groups. The pairwise deletion method was used to treat missing data. Univariate logistic regression analysis was used to examine the association of each variable with oxygen treatment or death while controlling for age. Additionally, multivariate logistic regression analysis was performed to control for confounding factors. All tests were two-tailed, and significance was assessed at *p* < 0.05. SPSS (version 23.0, IBM, Armonk, NY, USA) was used for the analyses.

## 3. Results

A total of 340 elderly patients with COVID-19 were enrolled in this study. Among these patients, 51 patients (15%) passed away, and 289 patients were recovered and were discharged. Except for two patients who died immediately after hospitalization, 119 patients (35.2%) were classified as severe pneumonia group received oxygen therapy, and 219 patients with mild pneumonia recovered without oxygen therapy.

The demographics and baseline characteristics between groups are presented in Table 1. The severe pneumonia group was older, had more male patients, and had shorter days from diagnosis to admission than the mild pneumonia group. The same findings were observed in the comparison between the deceased group and the survival group. The total hospital days were longer in the severe pneumonia group than in the mild pneumonia group. However, the total hospital days of the deceased group were shorter than those of the survival group. Among these 340 patients, 40 patients (11.8%) lived in the nursing facility and 84 patients (24.7%) reported ADL impairment. Overall, 244 of these patients (71.8%) had underlying diseases, and the most common underlying diseases were hypertension (*n* = 188, 55.3%) and diabetes (*n* = 106, 31.1%). Of these patients, 70 (20.6%) had chronic neurologic diseases, including stroke (*n* = 33, 9.7%) and dementia (*n* = 49, 14.4%).

There were more patients with ADL impairment and higher comorbidity scores in both the severe pneumonia group and deceased group. The respiratory rate and body temperature at admission were higher in the severe pneumonia group and deceased group. In the severe pneumonia group and the deceased group, more patients had pneumonia in the chest radiograph performed at admission than the control group.

Common symptoms in elderly patients with COVID-19 were fever (*n* = 172, 50.6%), cough (*n* = 129, 37.9%), sputum (*n* = 99, 29.1%), and dyspnea (*n* = 98, 28.8%), which were related to pneumonia.

In binary logistic regression analysis to assess the relationship between initial symptoms and prognosis, patients with initial dyspnea had a higher risk of severe pneumonia (OR: 7.71, 95% confidence interval (CI) 4.48–13.29, *p*-value < 0.001) and death (OR: 4.16, 95% CI 2.18–7.92, *p*-value < 0.001). Additionally, patients with initial cough had a higher risk of severe pneumonia (OR: 1.64, 95% CI 1.01–2.64, *p*-value = 0.004). Also, patients with nonspecific symptoms of myalgia were relatively common, and patients with fatigue had a high risk of severe pneumonia and death (OR: 7.89, 95% CI 1.59–39.07, *p*-value = 0.011; OR: 6.14, 95% CI 1.59–23.66, *p*-value = 0.008) (Table 2).

**Figure 1:**
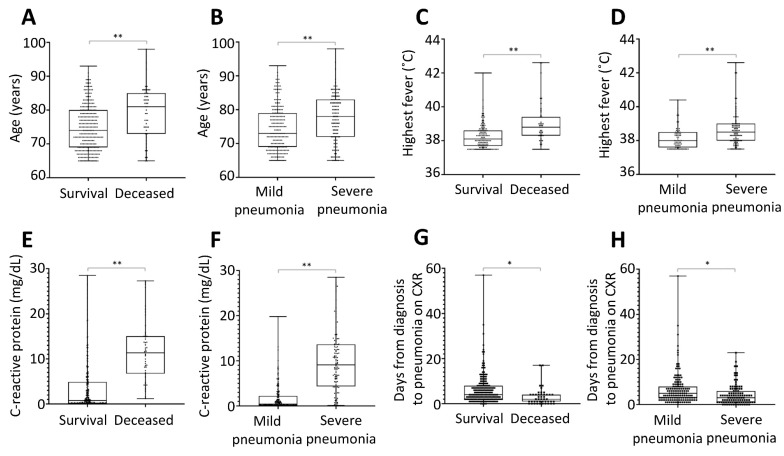
Potential predictors associated with poor prognosis in elderly patients with COVID-19 infection. The age, highest fever, C-reactive protein, and days from diagnosis to pneumonia on chest X-ray were significantly associated with poor outcomes, including death (**A**,**C**,**E**,**G**) and severe pneumonia (**B**,**D**,**F**,**H**). * *p* < 0.05; ** *p* < 0.001.

Comparison of laboratory findings and chest radiograph results was performed using ANCOVA while controlling for age. In the comparison analysis, lower lymphocytes, higher white blood cells (WBC), lower platelets, a lower level of albumin, higher ALT/AST, higher BUN, higher creatine, higher C-reactive protein (CRP), higher glucose, higher LDH, and higher aPTT were associated with severe pneumonia and death in elderly patients with COVID-19 (Figure 1 and Table S1).

The age-adjusted univariate logistic regression analysis showed that age, male sex, baseline ADL impairment, a comorbidity score of 2 or more, fever, infiltration at initial chest X-ray, and CRP at admission were associated with severe pneumonia and death of elderly patients with COVID-19 infection (Table 3 and Table 4).

Multivariate logistic regression analysis was performed to control for confounding factors. Premorbid baseline ADL impairment, fever during hospitalization, and increased CRP at admission were predictors of severe pneumonia in elderly patients with COVID-19 infection in multivariate logistic regression analysis (Table 3). Additionally, premorbid baseline ADL impairment, a comorbidity score of 2 or more, fever during hospitalization, and increased CRP at admission were predictive factors of death in elderly patients with COVID-19 infection after controlling for confounding factors (Table 4).

## 4. Discussion

The main findings of current study are that the prognosis of elderly patients with COVID-19 infection is associated with premorbid baseline ADL impairment, fever, and increased CRP at admission after adjusting for confounders. Additionally, patients’ premorbid baseline ADL impairment is significantly associated with severe pneumonia, requiring oxygen therapy, and higher mortality, rather than living in a nursing facility. Finally, fever during hospitalization and initial dyspnea are key symptoms in predicting severe pneumonia requiring oxygen therapy and mortality.

Age is a well-known, significant predictor of poor prognosis of COVID-19 infection [11,13,26]. In this study, as in many previous studies [14,18,19,27,28], age was observed among elderly COVID-19 patients over 65 years old as a strong risk factor. Since age is a very strong prognostic factor, we performed an analysis to adjust for age for all other variables.

Although age significantly increases the risk of COVID-19, in cases with underlying diseases, the elderly has been reported to be more susceptible to COVID-19 [15,16,29]. We also found that higher comorbidity is associated with poor prognosis. In previous studies, hypertension and diabetes were common underlying diseases associated with COVID-19 patients [2,30], and the prevalence of those underlying diseases in this study was similar or slightly higher than that of other studies [2,11]. In this study, there was a statistically significant difference in the prevalence of diabetes in both the severe pneumonia group and deceased group compared with the control group. Several studies have been published on the influence of diabetes in patients with COVID-19 [31,32]. In this study, 106 patients (30.9%) had diabetes. The elderly COVID-19 patients with diabetes required significantly more oxygen therapy than those without diabetes (*p* = 0.003). Additionally, intensive care unit care, ventilator care, and mortality were significantly higher in patients with diabetes than those without. (*p* = 0.043, *p* = 0.005, *p* < 0.001, respectively) (Supplementary Table S2). Therefore, in line with previous research [17], we suggest that the elderly COVID-19 patients with diabetes are more susceptible to COVID-19 infection and poor outcomes.

Among the common symptoms, initial dyspnea at admission and fever during hospitalization were strong risk factors for poor prognosis of both severe pneumonia and death in elderly patients with COVID-19, and these findings are consistent with previous studies [3,33]. Additionally, elderly patients with COVID-19 had higher clinical usefulness of nonspecific symptoms, such as myalgia and fatigue, than typical symptoms, such as cough and sputum, related with pneumonia.

In univariate logistic analysis, male sex, fever, increased CRP, and pneumonia on chest radiograph were reported as factors associated with the prognosis of elderly patients with COVID-19, which is similar to previous studies. Male gender is one of the risk factors for increased severity and mortality independent of age [29,34,35]. Whereas men and women have the same prevalence of disease [36], men with COVID-19 are more at risk for worse outcomes. This gender difference is probably due to differences in the expression level of the ACE2 receptor to which SARS-COV-2 binds [37,38].

Additionally, it is well established that CRP is elevated in patients with COVID-19 and is higher in patients with the severe disease than nonsevere [39]. Additionally, a positive association between level of the CRP and severity of COVID-19 infection was also reported [40]. Recently, a study showed that CRP could serve as a possible marker to predict the risk of worsening of nonsevere COVID-19 infection [41]. These studies have suggested that CRP level may be an early marker to anticipate risk for severe COVID-19 infection [42].

The prognosis of disease in elderly patients with COVID-19 infection is associated with the patient’s premorbid baseline ADL impairment rather than simply living in a nursing facility. In terms of mortality, the patient’s individual ADL impairment had a more significant impact on death than whether they lived in a nursing facility. The baseline ADL impairment investigated in this study seems to include not only cognitive ADL impairment, but both mental and physical ADL impairment. Based on these results, it may be necessary to consider premorbid baseline ADL impairment rather than simply identifying the location of the outbreak or residence to determine whether to hospitalize patients or place them in intensive care during a large-scale outbreak for an efficient healthcare system.

The older patients become and the more underlying diseases they have, the more likely they are to make a do-not-resuscitate (DNR) decision, which might be related to the consideration of their premorbid baseline ADL. However, the patients with DNR in this study received sufficient medical treatment, such as antiviral or antibiotic agents, oxygen supply, ventilator, dialysis, and even extracorporeal membrane oxygenation until the DNR decision at the time of exacerbation, as compared with patients without the DNR decision. Supplemental Table S3 shows the comparison of treatments in each group.

For patients in nursing facilities, the severity of the disease could be reduced through massive and intensive screening test [43,44]. By conducting a massive screening test, it was possible to reduce the incidence of severe pneumonia and death in elderly COVID-19 patients who lived in nursing facilities. Besides, patients with relatively mild symptoms among nursing facility patients may have been selectively included in this study due to the characteristics of participating hospitals. Only 40 of the 540 patients in a nursing facility outbreak, which occurred until 31 May 2020 in Daegu, were included in this study.

The duration from diagnosis to admission was short in both groups with poor prognosis, likely because of the hospitalization selection system in Daegu City, South Korea. The same findings were observed after including information from referral hospitals (Supplementary Table S4). During the outbreak, volunteer physicians identified symptoms, signs, and underlying diseases of patients with COVID-19 through a phone interview and selected patients who needed hospitalization urgently [24]. Also, patients with high medical requirements were distributed and hospitalized in several hospitals, and a referral system was operated efficiently. Since this healthcare system was effective, it allowed patients with COVID-19 in need of intensive treatment to be hospitalized with priority, resulting in reduced death and fatality rates.

This study has several strengths. First, this study included a relatively large number of elderly patients. Second, all clinical information up until death and clinical outcomes of patients were included in the study, since only patients with confirmed discharge or death were included in the study. To the best of our knowledge, the patients in the previous geriatric reports remained hospitalized at the time of publication [18,45]. Third, data on the presence of premorbid baseline ADL impairment, nursing facility residence, and dementia, which can affect the prognosis of elderly patients, were included. Fourth, a trained radiologist with sufficient experience in COVID-19 pneumonia performed consistent chest radiograph readings.

This study has several limitations. It was a retrospective and multicenter study, and some relevant data, such as smoking history, were incomplete and no formal scale was used to evaluate patient’s ADL. Also, some test results, such as chest CT, IL-6, and pro-calcitonin, known as prognostic factors for COVID-19 [18], could not be included in the analysis because of missing data. Additionally, patients were only included from three hospitals in Daegu. Some patients were referred to our hospital, and some of our patients were transferred to other hospitals. Thus, the data of patients before referral or after transfer were not included. Finally, information such as the type or duration of oxygen therapy was not included in this study. In the future study, more detailed clinical information on oxygen therapy may provide additional knowledge about the prognostic factors for COVID-19 infection.

## 5. Conclusions

In conclusion, this study showed that premorbid ADL impairment, fever during hospitalization, and initial CRP were associated with poor prognosis in elderly patients with COVID-19. In particular, premorbid ADL impairment in elderly patients had a stronger effect on the poor prognosis of COVID-19 than other variables. To control COVID-19 infection in elderly patients, it is necessary to understand and screen these indicators of poor prognosis, so that patients who have these problems can be treated with fast and quick selected strategies.

## Figures and Tables

**Table 1:** Demographics and baseline characteristics between groups.

Characteristics	Severe Pneumonia (*n* = 119)	Mild Pneumonia (*n* = 219)	*p*-Value	Deceased (*n* = 51)	Survival (*n* = 289)	*p*-Value
Age, years	77.32 (7.25)	74.39 (6.70)	<0.001	79.08 (7.81)	74.76 (6.70)	<0.001
Sex, male, *n* (%)	61 (51.3)	68 (31.1)	<0.001	29 (56.9)	101 (34.9)	0.003
Days from symptom onset to diagnosis	2.92 (1.98–3.87)	2.69 (2.01–3.38)	0.704	2.85 (1.47–4.22)	2.74 (2.14–3.35)	0.895
Days from symptom onset to admission	5.05 (3.47–6.63)	8.40 (7.33–9.47)	<0.001	3.82 (1.45–6.2)	7.90 (6.94–8.85)	0.002
Days from diagnosis to admission	2.88 (1.75–4.01)	5.84 (5.10–6.59)	<0.001	1.55 (−0.20–3.30)	5.42 (4.76–6.08)	<0.001
Total hospital days	27.89 (25.2–30.59)	24.47 (22.49–26.44)	0.050	16.57 (12.50–20.63)	27.10 (25.42–28.78)	<0.001
Nursing facility living	19 (16.0)	21 (9.6)	0.083	11 (21.6)	29 (10.0)	0.018
ADL impairment	56 (47.1)	26 (11.9)	<0.001	35 (68.6)	49 (17.0)	<0.001
Smoking	8 (6.1)	13 (13.7)	0.051	6 (13.6)	15 (8.1)	0.253
Comorbidity			0.001			<0.001
2	60 (50.4)	65 (29.7)		34 (66.7)	92 (31.8)	
1	33 (27.7)	84 (38.4)		10 (19.6)	108 (37.4)	
0	26 (21.8)	70 (32.0)		7 (13.7)	89 (30.8)	
DM	49 (41.5)	56 (25.7)	0.003	28 (54.9)	78 (27.2)	<0.001
HTN	74 (62.7)	113 (51.8)	0.055	34 (66.7)	154 (53.7)	0.085
CHF/CAD	14 (11.9)	26 (11.9)	0.987	7 (13.7)	34 (11.9)	0.705
Lung disease	11 (9.2)	14 (6.4)	0.345	8 (15.7)	17 (5.9)	0.036
CKD	7 (5.9)	4 (1.8)	0.044	4 (7.8)	7 (2.4)	0.067
Malignant disorder	11 (9.3)	10 (4.6)	0.089	6 (11.8)	15 (5.2)	0.107
Dementia	24 (20.3)	25 (11.5)	0.028	13 (25.5)	36 (12.5)	0.016
Body mass index, kg/m^2^	23.58 (22.86–24.31)	23.07 (22.48–23.66)	0.280	23.35 (22.17–24.52)	23.26 (22.76–23.76)	0.895
Initial systolic BP, mmHg	135.32 (131.44–139.19)	145.82 (142.95–148.69)	<0.001	133.32 (127.13–139.51)	143.58 (141.07–146.10)	0.003
Initial diastolic BP, mmHg	75.86 (73.57–78.14)	83.04 (81.35–84.73)	<0.001	75.71 (72.02–79.40)	81.30 (79.80–82.80)	0.006
Initial HR, per min	90.06 (87.04–93.09)	87.37 (85.12–89.61)	0.160	91.33 (86.56–96.10)	87.81 (85.87–89.75)	0.182
Initial RR, per min	22.84 (22.22–23.46)	19.81 (19.36–20.26)	<0.001	23.37 (22.33–24.40)	20.47 (20.06–20.88)	<0.001
Initial body temperature, °C	37.11 (36.99–37.23)	36.80 (36.71–36.89)	<0.001	37.08 (36.89–37.27)	36.88 (36.80–36.96)	0.059
CXR infiltration at admission	98 (82.4)	129 (58.9)	<0.001	43 (86.0)	185 (64.0)	0.002

All continuous variables were adjusted for age using analysis of covariance (ANCOVA). Data are presented as the LS (least squares) means (IQR, interquartile range) or *n* (%), except age, and age data are presented as the means (SD). ADL, activities of daily living; BP, blood pressure; HR, heart rate; RR, respiratory rate; CXR, chest X-ray.

**Table 2:** Binary logistic regression analysis predicting odds of symptoms in patients with COVID-19.

Symptoms	Pneumonia					Death				
	Severe Pneumonia (*n* = 119)	Mild Pneumonia (*n* = 219)	OR	95% CI	*p*-Value	Deceased (*n* = 51)	Survival (*n* = 289)	OR	95% CI	*p*-Value
Fever	97 (81.5)	75 (34.2)	8.54	4.92–14.81	<0.001	43 (87.8)	129 (44.6)	8.62	3.52–21.11	<0.001
Cough	51 (43.6)	78 (35.8)	1.64	1.01–2.64	0.004	112 (39.0)	17 (34.0)	0.98	0.51–1.88	0.947
Sputum	39 (33.3)	60 (27.5)	1.67	1.00–2.79	0.051	83 (28.9)	16 (32.0)	1.63	0.82–3.24	0.168
Sore throat	12 (10.3)	25 (11.5)	1.12	0.53–2.37	0.775	34 (11.9)	3 (6.0)	0.65	0.19–2.26	0.495
Rhinorrhea	11 (9.4)	29 (13.3)	0.72	0.34–1.52	0.386	36 (12.5)	4 (8.0)	0.66	0.22–1.99	0.456
Chest pain	7 (6.0)	7 (3.2)	2.14	0.71–6.44	0.175	11 (3.8)	4 (8.0)	2.73	0.79–9.43	0.112
Myalgia	24 (20.5)	56 (25.7)	0.93	0.53–1.63	0.799	75 (26.1)	6 (12.0)	0.52	0.21–1.30	0.159
Fatigue	8 (6.8)	2 (0.9)	7.89	1.59–39.07	0.011	5 (10.0)	5 (1.7)	6.14	1.59–23.66	0.008
Dyspnea	65 (55.6)	33 (15.1)	7.71	4.48–13.29	<0.001	70 (24.4)	28 (56.0)	4.16	2.18–7.92	<0.001
Headache	11 (9.4)	49 (22.5)	0.44	0.21–0.89	0.022	58 (20.2)	3 (6.0)	0.35	0.10–1.20	0.095
Nausea/vomiting	3 (2.6)	6 (2.8)	0.98	0.23–4.10	0.975	3 (6.0)	7 (2.4)	3.20	0.75–13.71	0.116
Diarrhea	11 (9.4)	23 (10.6)	1.01	0.47–2.20	0.976	33 (11.5)	2 (4.0)	0.39	0.09–1.70	0.208

Logistic regression analysis was performed to control for age. The data are presented as *n* (%). Fever (≥37.5 °C) was measured during hospitalization and other symptoms were checked at admission. OR, odds ratio; CI, confidence interval.

**Table 3:** Univariate and multivariate logistic regression analysis of predictive factors for severe pneumonia with COVID-19.

Variables	Univariate			Multivariate		
	Adjusted OR	95% CI	*p*-Value	OR	95% CI	*p*-Value
Age	1.06	1.03–1.10	<0.001			
Sex	2.38	1.49–3.81	<0.001			
Nursing facility living	0.67	0.34–1.33	0.255			
ADL impairment	5.84	3.27–10.42	<0.001	5.33	2.42–11.73	<0.001
Comorbidity	2.15	1.34–3.44	0.002	1.97	0.96–4.01	0.064
Dementia	1.38	0.71–2.66	0.340			
Fever	8.54	4.92–14.81	<0.001	3.20	1.52–6.74	0.002
Initial CXR infiltration	3.37	1.94–5.85	<0.001	2.32	1.02–5.27	0.044
Initial CRP	1.45	1.33–1.58	<0.001	1.33	1.21–1.45	<0.001

All variables were adjusted for age in univariate logistic regression analysis except age. OR, odds ratio; CI, confidence interval; ADL, activities of daily living; CXR, chest X-ray; CRP, C-reactive protein.

**Table 4:** Univariate and multivariate logistic regression analysis of predictive factors for death with COVID-19.

Variables	Univariate			Multivariate		
	Adjusted OR	95% CI	*p*-Value	OR	95% CI	*p*-Value
Age	1.09	1.04–1.14	<0.001	1.06	1.00–1.13	0.063
Sex	2.58	1.38–4.81	0.003			
Nursing facility living	1.90	0.85–4.25	0.119			
ADL impairment	8.89	4.37–18.10	<0.001	7.13	2.93–17.40	<0.001
Comorbidity	3.68	1.93–7.02	<0.001	3.28	1.43–7.54	0.005
Dementia	1.43	0.66–3.13	0.368			
Fever	8.62	3.52–21.11	<0.001	3.15	1.10–9.03	0.032
Initial CXR infiltration	3.55	1.52–8.30	0.003			
Initial CRP	1.20	1.14–1.27	<0.001	1.18	1.11–1.26	<0.001

All variables were adjusted for age in univariate logistic regression analysis except age. OR, odds ratio; CI, confidence interval; ADL, activities of daily living; CXR, chest X-ray; CRP, C-reactive protein.

## Supplementary Materials

The following are available online at https://www.mdpi.com/2077-0383/9/12/3932/s1, Table S1: Comparison of laboratory test results and chest radiograph finding between groups. Table S2: Comparison between patients with diabetes and without diabetes. Table S3: Comparison of treatments between groups. Table S4: Duration to admission in both groups (including information of referral hospitals). Table S5: Comparison between deceased patients and recovered patients among the patients with severe pneumonia.
