# Supplementary material for: Prognostic Factors of COVID-19 Infection in Elderly Patients: A Multicenter Study
Source: J Clin Med. 2020 Dec 4;9(12):3932. doi: 10.3390/jcm9123932 (PMC7761972; doi:10.3390/jcm9123932)
Supplement: Supplementary file 1 [file jcm-09-03932-s001.pdf]

Table S1. Comparison of laboratory test results and chest radiograph finding between groups.

	Severe pneumonia (N=119)	Mild pneumonia (N=219)	P-value	Deceased (N=51)	Survival (N=289)	P-value
**Initial laboratory results**						
Hemoglobin, g/L	12.14(11.85-12.43)	12.09(11.87-12.3)	0.760	12.37(11.92-12.82)	12.05(11.87-12.23)	0.196
Hematocrit, %	36.29(35.43-37.15)	36.4(35.76-37.03)	0.850	37.34(36-38.68)	36.16(35.61-36.71)	0.113
Lymphocytes, $\times10^9$ cells/L	16.02(14.18-17.87)	27.33(25.97-28.69)	<0.001	14.02(10.92-17.12)	24.91(23.66-26.17)	<0.001
Platelets, $\times10^9$ cells/L	204.57(187.56-221.58)	240.93(228.47-253.4)	<0.001	184.36(157.62-211.11)	235.55(224.75-246.36)	0.001
WBCs, $\times10^9$ cells/L	7.33(6.84-7.81)	5.88(5.52-6.24)	<0.001	8.6(7.85-9.35)	6.02(5.71-6.32)	<0.001
Albumin, mg/L	3.33(3.24-3.41)	3.83(3.77-3.9)	<0.001	3.26(3.11-3.4)	3.72(3.66-3.78)	<0.001
ALT, U/L	46.1(30.59-61.61)	24.72(13.4-36.04)	0.030	47.02(22.37-71.68)	29.75(19.88-39.62)	0.204
AST, U/L	79.05(50.49-107.61)	28.47(7.54-49.39)	0.010	77.81(32.49-123.13)	40.93(22.62-59.24)	0.141
BUN, mg/dL	24.16(21.98-26.34)	17.06(15.46-18.66)	<0.001	28.59(25.18-32.01)	18.03(16.65-19.4)	<0.001
Creatinine, μmol/L	1.21(1.06-1.37)	0.9(0.79-1.01)	<0.001	1.36(1.12-1.6)	0.95(0.85-1.05)	0.002
C-reactive protein, mg/dL	10.09(9.19-10.99)	1.8(1.1-2.5)	<0.001	11.99(10.37-13.6)	3.64(2.97-4.32)	<0.001
Blood glucose, mg/dL	164.82(152.03-177.61)	130.21(121.12-139.29)	<0.001	170.64(150.1-191.17)	137.36(129.31-145.4)	0.003
Sodium, mmol/L	136.41(135.56-137.25)	139.64(139.02-140.26)	<0.001	136.14(134.76-137.52)	138.9(138.35-139.45)	<0.001
Potassium, mmol/L	4.08(3.97-4.19)	4.22(4.14-4.3)	0.060	4.13(3.95-4.31)	4.18(4.1-4.25)	0.677
Chloride, mmol/L	100.02(89.13-110.9)	104.63(97.18-112.08)	0.500	101.47(84.16-118.78)	103.39(96.84-109.95)	0.839
LDH, U/L	668.16(625.17-711.15)	471.73(442.9-500.56)	<0.001	749.96(679.8-820.12)	502.64(476.74-528.55)	<0.001
Total bilirubin, mg/dL	0.75(0.62-0.88)	0.57(0.48-0.67)	0.030	0.65(0.45-0.86)	0.63(0.55-0.72)	0.84
aPTT	31.84(30.69-32.98)	28.48(27.69-29.26)	<0.001	33.44(31.59-35.29)	29(28.31-29.69)	<0.001
PT, sec	14.48(13.33-15.63)	12.24(11.44-13.04)	<0.001	15.91(14.08-17.74)	12.53(11.83-13.23)	0.001
PT, INR	1.22(1.07-1.38)	1.08(0.97-1.19)	0.140	1.34(1.09-1.59)	1.09(1-1.19)	0.067
**Chest radiograph findings**						
Days from diagnosis to pneumonia on CXR	4.40(3.16-5.64)	6.78(5.76-7.79)	0.004	3.04(1.15-4.93)	6.4(5.54-7.26)	0.002

Data are presented as the LS mean (IQR, interquartile range) or n (%). Age adjustment was performed using ANCOVA and logistic regression test. WBC, white blood cells; ALT, alanine aminotransferase; AST, aspartate aminotransferase; BUN, blood urea nitrogen; LDH, lactate dehydrogenase; aPTT, Activated partial thromboplastin time; PT, prothrombin time; CXR, chest X-ray.

Table S2. Comparison between patients with diabetes and without diabetes.

	Diabetes (N=106)	Non-Diabetes (N=232)	P-value
Age, years	75.82±6.64	75.15±7.2	0.417
Sex, male, n (%)	50(47.2)	78(33.6)	0.017
Days from symptom onset to diagnosis	2.68±3.78	2.81±4.65	0.820
Days from symptom onset to admission	7.49±7.8	7.2±6.48	0.773
Days from diagnosis to admission	4.96±6.71	4.88±4.83	0.905
Total hospital days	23.98±15.58	26.13±14.54	0.218
Nursing facility living	14(13.2)	26(11.2)	0.597
ADL impairment	27(25.5)	57(24.6)	0.859
Smoking	5(6.6)	16(10.5)	0.331
**Premorbid underlying disease**			
HTN	84(79.2)	104(44.8)	<0.001
CHF/CAD	15(14.2)	26(11.2)	0.442
Lung disease	7(6.6)	17(7.3)	0.810
Chronic kidney disease	2(1.9)	9(3.9)	0.338
Malignancy	3(2.8)	18(7.8)	0.080
Dementia	14(13.2)	35(15.1)	0.649
Body mass index, kg/m^2	23.56±2.86	23.16±3.52	0.442
**Initial vital sign**			
Initial systolic BP, mmHg	141.37±19.97	142.54±22.8	0.653
Initial diastolic BP, mmHg	79.21±14.06	81.16±12.86	0.213
Initial HR, per min	89±16.87	87.93±16.45	0.586
Initial RR, per min	20.99±4.29	20.83±3.33	0.706
Initial body temperature, ℃	36.93±0.7	36.9±0.64	0.719
**Symptoms and sign**			
Highest fever during admission	38.73±1.13	38.31±0.77	0.014
Fever	56(53.3)	115(49.8)	0.546
Cough	39(37.1)	90(39.0)	0.751
Sputum	29(27.6)	70(30.3)	0.617
Sore throat	8(7.6)	29(12.6)	0.180
Rhinorrhea	9(8.6)	31(13.4)	0.203
Chest pain	5(4.8)	10(4.3)	0.859
Myalgia	20(19.0)	61(26.4)	0.144
Fatigue	3(2.9)	7(3.0)	0.931
Dyspnea	32(30.5)	66(28.6)	0.722
Headache	18(17.1)	43(18.6)	0.746
Nausea/vomiting	1(1.0)	9(3.9)	0.181
Diarrhea	10(9.5)	25(10.8)	0.718
**Initial laboratory results**			
Hemoglobin, g/L	12.03±1.79	12.12±1.56	0.643

Hematocrit, %	36.18±5.4	36.38±4.59	0.726
Lymphocytes, ×10^9 cells/L	20.82±10.88	24.54±11.99	0.007
Platelets, ×10^9 cells/L	228.78±100.28	227.01±92.17	0.874
WBCs, ×10^9 cells/L	7.02±3.16	6.11±2.56	0.005
Albumin, mg/L	3.59±0.59	3.68±0.54	0.169
ALT, U/L	27.47±30.97	34.48±100.44	0.486
AST, U/L	38.07±38.09	50.21±189.16	0.515
BUN, mg/dL	20.56±13.11	19.13±12.74	0.348
Creatinine, μmol/L	1.08±0.64	0.98±0.96	0.327
C-reactive protein, mg/L	6.65±7.22	4.11±5.59	0.003
Blood glucose, mg/dL	185.29±96.64	121.26±38.27	<0.001
Sodium, mmol/L	137.58±4.48	138.92±4.97	0.019
Potassium, mmol/L	4.28±0.6	4.12±0.62	0.023
Chloride, mmol/L	99.23±4.07	105.01±66.28	0.387
LDH, U/L	559.21±253.62	520.18±215.98	0.169
Total bilirubin, mg/dL	0.63±0.77	0.63±0.7	0.974
aPTT	29.37±3.74	29.67±6.72	0.615
PT, sec	12.23±1.31	13.32±7.11	0.033
PT, INR	1.03±0.12	1.17±0.96	0.032
**Chest radiograph**			
Days from diagnosis to pneumonia on CXR	4.61±4.41	6.37±7.06	0.017
**Treatment and Prognosis**			
**Medication**	104(99.0)	227(98.3)	0.585
Anti-viral	79(75.2)	147(63.6)	0.036
Anti-malarial	65(61.9)	142(61.5)	0.940
Anti-bacterial	92(87.6)	196(84.8)	0.501
Steroid	33(31.4)	38(16.5)	0.002
Oxygen therapy	49 (46.7%)	69 (29.9%)	0.003
Intensive care unit	26 (24.8%)	36 (15.5%)	0.043
Ventilator care	26 (24.8%)	29 (12.5%)	0.005
ECMO	5 (4.8%)	3 (1.3%)	0.114
Dialysis	4(3.8)	7(3.0)	0.745
Death	28 (26.4%)	23 (9.9%)	<0.001

Table S3. Comparison of treatments between groups.

	Severe pneumonia (N=119)	Mild pneumonia (N=219)	P-value	Deceased (N=51)	Survival (N=289)	P-value
**Medication**	119(100.0)	214(97.7)	0.097	49(100%)	284(98.3%)	0.350
Anti-viral	95(79.8)	133(60.7)	<0.001	42(85.7%)	186(64.4%)	0.003
Anti-malarial	78(65.5)	130(59.4)	0.264	33(67.3%)	175(60.6%)	0.366
Anti-bacterial	117(98.3)	173(79.0)	<0.001	49(100%)	241(83.4%)	0.002
Steroid	58(48.7)	14(6.4)	<0.001	28(57.1%)	44(15.2%)	<0.001
Intensive care unit	57(47.9)	4(1.8)	<0.001	28(56%)	34(11.8%)	<0.001
Ventilator care	56(47.1)	0	<0.001	40(80%)	16(5.5%)	<0.001
ECMO	8(6.7)	0	<0.001	7(14%)	1(0.3%)	<0.001
Dialysis	10(8.4)	1(0.5)	<0.001	8(16%)	3(1%)	<0.001

Table S4. Duration to admission in both groups (including information of referral hospitals).

	Severe pneumonia (N=119)	Mild pneumonia (N=219)	P-value	Deceased (N=51)	Survival (N=289)	P-value
Days from symptom onset to admission	5.67±6.19	8.35±7.19	0.002	4.69±5.03	7.97±7.19	0.019
Days from diagnosis to admission	3.15±3.85	5.91±6.10	<0.001	2.25±2.78	5.47±5.84	0.001

Table S5. Comparison between deceased patients and recovered patients among the patients with severe pneumonia.

	Deceased (N=49)	Recovered (N=70)	P-value
Age, years	79.33±7.78	75.91±6.56	0.011
Sex, male, n (%)	28(57.1%)	33(47.1%)	0.283
Days from symptom onset to diagnosis	2.73±3.88	2.89±4.62	0.856
Days from symptom onset to admission	3.56±4.61	5.90±5.76	0.066
Days from diagnosis to admission	1.49±1.74	3.77±3.46	<0.001
Total hospital days	17.67±15.33	34.86±16.16	<0.001
Nursing facility living	11(22.4%)	8(11.4%)	0.106
ADL impairment	33(67.3%)	23(32.9%)	<0.001
Smoking	36(85.7%)	46(86.8%)	0.879
Comorbidity			0.008
2	33(67.3%)	27(38.6%)	
1	9(18.4%)	24(34.3%)	
0	7(14.3%)	19(27.1%)	
**Premorbid underlying disease**			
DM	27(55.1%)	22(31.9%)	0.012
HTN	33(67.3%)	41(59.4%)	0.380
CHF/CAD	6(12.2%)	8(11.6%)	0.914
Lung disease	8(16.3%)	3(4.3%)	0.049
Chronic kidney disease	4(8.2%)	3(4.3%)	0.447
Malignancy	6(12.2%)	5(7.2%)	0.522
Dementia	13(26.5%)	11(15.9%)	0.159
Body mass index, kg/m^2	22.91±3.69	23.79±3.41	0.284
**Initial vital sign**			
Initial systolic BP, mmHg	132.45±24.46	136.69±21.79	0.323
Initial diastolic BP, mmHg	74.35±15.00	75.91±13.48	0.552
Initial HR, per min	90.43±21.14	89.10±18.10	0.714
Initial RR, per min	23.51±5.49	22.49±4.56	0.275
Initial body temperature, °C	37.04±0.87	37.12±0.79	0.623
**Symptoms and sign**			
Fever	43(87.8%)	54(77.1%)	0.142
Cough	17(35.4%)	34(49.3%)	0.137
Sputum	16(33.3%)	23(33.3%)	>0.999
Sore throat	3(6.3%)	9(13.0%)	0.355
Rhinorrhea	4(8.3%)	7(10.1%)	>0.999
Chest pain	3(6.3%)	4(5.8%)	>0.999
Myalgia	5(10.4%)	19(27.5%)	0.024
Fatigue	5(10.4%)	3(4.3%)	0.270
Dyspnea	28(58.3%)	37(53.6%)	0.614
Headache	2(4.2%)	9(13.0%)	0.196
Nausea/vomiting	2(4.2%)	1(1.4%)	0.567

Diarrhea	1(2.1%)	10(14.5%)	0.026
**Initial laboratory results**			
Hemoglobin, g/L	12.17±2.09	11.92±1.81	0.487
Hematocrit, %	36.84±6.59	35.4±5.24	0.189
Lymphocytes, $\times 10^9$ cells/L	12.83±8.34	17.37±9.48	0.008
Platelets, $\times 10^9$ cells/L	181.57±81.31	218.06±98.95	0.036
WBCs, $\times 10^9$ cells/L	8.77±4.56	6.49±2.85	0.003
Albumin, mg/L	3.18±0.48	3.37±0.40	0.019
ALT, U/L	46.02±127.02	45.04±147.15	0.970
AST, U/L	80.22±156.57	79.39±316.17	0.986
BUN, mg/dL	30±20.84	21.31±13.83	0.013
Creatinine, µmol/L	1.45±0.97	1.12±1.40	0.165
C-reactive protein, mg/L	11.82±6.85	8.75±7.04	0.021
Blood glucose, mg/dL	165.73±86.88	159.59±91.93	0.725
Sodium, mmol/L	136.15±6.21	136.63±5.39	0.654
Potassium, mmol/L	4.14±0.87	4.05±0.70	0.521
Chloride, mmol/L	99.66±5.92	98.95±5.77	0.550
LDH, U/L	746.79±409.52	613.81±244.16	0.077
Total bilirubin, mg/dL	0.63±0.33	0.81±0.99	0.244
aPTT	33.40±9.42	30.81±6.41	0.104
PT, sec	15.89±12.52	13.54±5.64	0.265
PT, INR	1.36±1.11	1.15±0.50	0.263
**Chest radiograph**			
Days from diagnosis to pneumonia on CXR	3.11±3.75	5.35±4.82	0.013
